# *N-*nonyloxypentyl-L-deoxynojirimycin reduces *Stenotrophomonas maltophilia* virulence *in vitro* and in *Galleria mellonella* infection model

**DOI:** 10.3389/fmicb.2026.1835243

**Published:** 2026-05-29

**Authors:** Maria Stabile, Anna Esposito, Antonella Migliaccio, Leandra Sepe, Rosaria Artiano, Antonella Ricca, Vita Dora Iula, Raffaele Zarrilli, Annalisa Guaragna, Eliana De Gregorio

**Affiliations:** 1 Department of Molecular Medicine and Medical Biotechnology, University of Naples Federico II, Naples, Italy; 2 Department of Public Health, School of Medicine and Surgery, University of Naples Federico II, Naples, Italy; 3 Department of Chemical Sciences, University of Naples Federico II, Naples, Italy; 4 Department of Laboratory Medicine, U.O.C Patologia Clinica, Ospedale del Mare-ASL Napoli 1 Centro, Naples, Italy

**Keywords:** adhesion inhibition, anti-virulence drug, biofilm eradication, iminosugars, L-deoxynojirimycin, proteases reduction

## Abstract

**Introduction:**

*Stenotrophomonas maltophilia* is an opportunistic pathogen able to resist multiple classes of antibiotics and form robust biofilms, making it difficult to treat.

**Methods:**

The antimicrobial and anti-virulence activity of the iminosugar *N*-nonyloxypentyl-L-deoxynojirimycin (L-NPDNJ) against *S. maltophilia* was evaluated by *in vitro* and *in vivo* models.

**Results and discussion:**

L-NPDNJ exhibited weak bactericidal activity but showed additive effects when used in combination with amikacin and tobramycin, reducing their minimum inhibitory concentrations by two- to threefold. At sub-inhibitory concentrations, L-NPDNJ was found to significantly modulate the expression of genes involved in antibiotic resistance, biofilm regulation, and virulence, including those encoding efflux pumps, global regulators, and extracellular proteases. These transcriptional changes were found to be associated with significant phenotypic effects. At sub-inhibitory concentrations, L-NPDNJ reduced cell surface hydrophobicity by up to 90%, impaired adhesion to abiotic surfaces and to human lung epithelial cells by over 90%, and disrupted preformed biofilms, decreasing biofilm biomass and metabolic activity by approximately 70%. In addition, L-NPDNJ has been shown to enhance macrophage phagocytosis and reduce intracellular bacterial survival, while improving epithelial cell viability. Notably, pretreatment of *S. maltophilia* cells with L-NPDNJ led to a substantial reduction in bacterial virulence in the *Galleria mellonella* infection model in a dose-dependent manner.

**Conclusion:**

Collectively, these findings demonstrate that L-NPDNJ exerts a broad anti-virulence effect against *S. maltophilia* and suggest its potential application as an adjuvant strategy to improve the treatment of infections caused by this multidrug-resistant pathogen.

## 1 Introduction

*Stenotrophomonas maltophilia* has emerged as an important opportunistic pathogen on a global scale, with a worrying trend toward multidrug resistance (Trifonova and Strateva, 2019; Brooke, 2021). This environmental bacterium can cause both nosocomial and community-acquired infections, and its genetic heterogeneity among animal, environmental, and human strains suggests a high degree of adaptability (Di Nocera et al., 2013; Mercier-Darty et al., 2020). *S. maltophilia* is a frequent cause of nosocomial infections in healthcare, especially among immunocompromised patients or those with indwelling medical devices (Kanderi et al., 2020; Huang et al., 2024). It has been found to be associated to infections of the respiratory system, bloodstream, urinary tract and wounds (Brooke, 2021), particularly in patients with cystic fibrosis (CF), chronic lung disease, cancer, organ transplantation, and immunosuppression (Cuthbertson et al., 2020). These infections are frequently severe, with mortality rates ranging from 21% to 69% (Insuwanno et al., 2020; Kanchanasuwan et al., 2022). A 10-year study from 2008 to 2017 revealed a striking 162% increase in *S. maltophilia* isolate incidence among patients with lower respiratory tract infections (Gajdács and Urbán, 2019). The ability of *S. maltophilia* to persist in the host and to cause disease depends on its capacity to resist multiple antibiotics, including β-lactams, aminoglycosides, and fluoroquinolones (Gil-Gil et al., 2020; Cruz-Córdova et al., 2020). *S. maltophilia* biofilm-associated infections are often chronic and recurrent, resulting in longer hospital admissions, higher morbidity and healthcare costs (Brooke, 2021; Flores-Treviño et al., 2019). In addition to the formation of biofilms, *S. maltophilia* possesses a complex arsenal of virulence factors that contribute to the damage of host tissue and immune evasion. These include extracellular enzymes (proteases, hemolysins, siderophores) and surface structures (LPS, fimbriae, flagella) that are essential for adhesion, motility, and host interaction (Bhaumik et al., 2024; Mikhailovich et al., 2024).

Given the intrinsic and acquired resistance of *S. maltophilia* to multiple classes of antibiotics, alternative therapeutic approaches are urgently needed (Monardo et al., 2025). Anti-virulence therapy has recently emerged as a promising strategy that aims to disarm rather than kill the pathogen, thereby minimizing selective pressure and the development of resistance (Dehbanipour and Ghalavand, 2022). These therapies target specific bacterial-host interactions or virulence mechanisms (such as quorum sensing, adhesion, and protease secretion), reducing the ability of pathogens to cause disease and allowing the host immune system to effectively clear the infection (Calvert et al., 2018; Lu et al., 2025; Smith et al., 2025).

**Figure 1:**
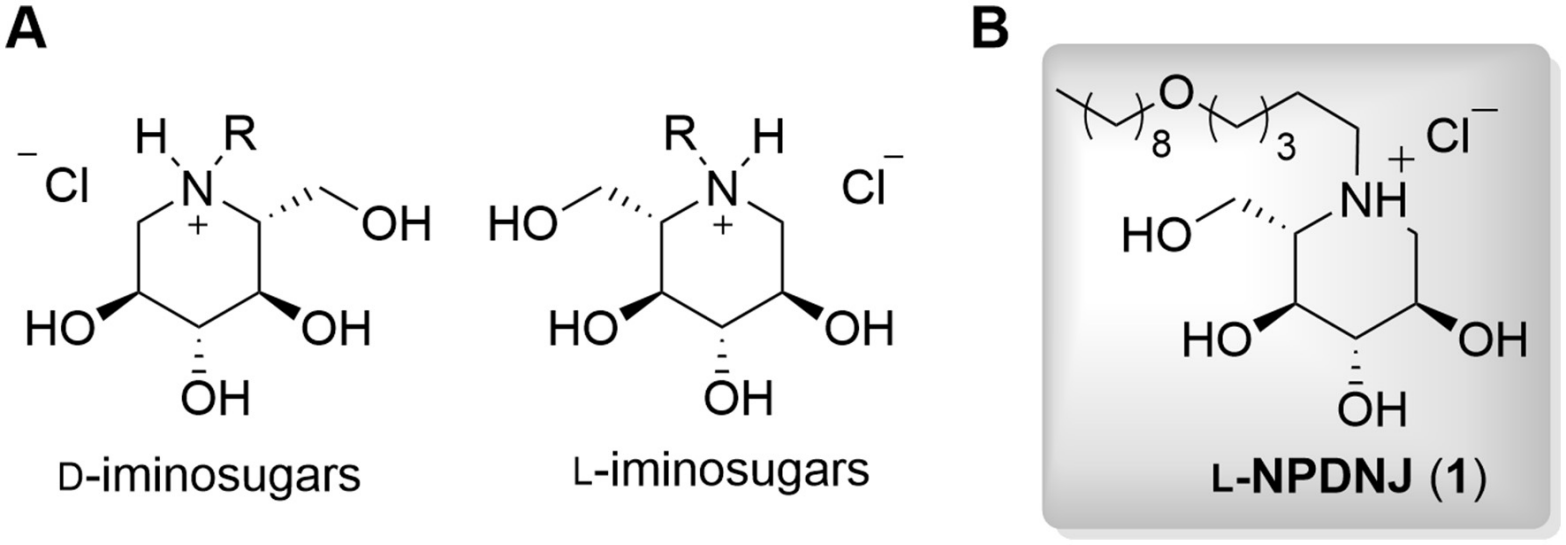
**(A)** *N*-substituted D- and L-iminosugars. **(B)** L-NPDNJ (*N*-nonyloxypentyl-L-deoxynojirimycin).

Iminosugars are natural or synthetic sugar analogs well-known for their broad-spectrum pharmacological properties (Esposito et al., 2020a). Over the years these glycomimetics have proved to be effective candidates in the management of several diseases including diabetes, malignancies and genetic disorders (Esposito et al., 2020a; Fiaux et al., 2005; De Pasquale et al., 2023; Matassini et al., 2023). Interestingly, iminosugars have also emerged as promising and innovative tools against bacterial infections (Strus et al., 2016; Esposito et al., 2020b; Kozień et al., 2022, 2024; Sluga et al., 2024). The broad-spectrum activity of iminosugars is due to their ability to interact with carbohydrate-processing enzymes, that are involved in several biological processes. However, this is responsible for their limited *in vivo* selectivity that often hampers their progress into clinics. Over the years, many changes have been proposed in the chemical structure of iminosugars for balancing biological activity and toxicity with promising results obtained by modification in the configuration of iminosugar core stereocenters. In the context we recently underscored the promising potential of unnatural L-gluco-configured iminosugars (Figure 1A). Particularly, some *N*-substituted L-deoxynojirimycin (DNJ) revealed interesting pharmacological properties in rare diseases such as Pompe, mucopolisaccharidosis and CF, associated with high enzymatic selectivity (De Pasquale et al., 2023; De Fenza et al., 2019; Esposito et al., 2024). In addition, some candidates have also shown promising antibacterial, antibiofilm and anti-virulence activity. Particularly, when evaluated against the major human Gram-positive pathogen *Staphylococcus aureus*, the iminosugar L-NPDNJ (*N*-nonyloxypentyl-L-DNJ, Figure 1B), a non-toxic compound for eukaryotic cells, was found as the most active compound among the series. L-NPDNJ has been shown to inhibit planktonic and sessile growth of *S. aureus*, as well as the expression of its major virulence genes (De Gregorio et al., 2020). In addition, it has been demonstrated to enhance the activity of antibiotics against methicillin-resistant strains (De Gregorio et al., 2020). The goal of this study was to assess the antimicrobial and anti-virulence properties of L-NPDNJ against *S. maltophilia* strains using *in vitro* and *in vivo* methods.

## 2 Materials and methods

### 2.1 Chemistry

All commercially available reagents and solvents were purchased at the highest degree of purity (Merck, Alfa Aesar, VWR) and used without further purification. Thin layer chromatography (F254 Merck silica gel plates) was employed to examine the reaction course with exposure to ultraviolet radiation (254 nm), iodine vapor, spraying with 5% ethanolic sulfuric acid solution and spraying ninhydrin. Purification of intermediates and final product was performed by column chromatography with silica gel (70–230 mesh, Merck Kiesegel 60). Compounds characterization was accomplished by NMR analysis using a Bruker AVANCE spectrometer (equipped with TOPSPIN) operating at 400 MHz (^1^H NMR) and 100 MHz (^13^C NMR) (see Supplementary Figures S1, S2). L-NPDNJ was synthesized and characterized according to the procedure previously reported (De Fenza et al., 2019). Optical rotation was measured at 25 ± 2 °C in methanol. Purity of L-NPDNJ was ≥95% as assessed by CHNS analysis and quantitative 1D 1H NMR (qHNMR) (De Pasquale et al., 2023; De Fenza et al., 2019).

### 2.2 Bacterial strains and growth conditions

A total of nine *S. maltophilia* clinical isolates, each given a unique ID and anonymized, as well as the reference strain K279a, were used in this study (Roscetto et al., 2015). Strains were cultured under aerobic conditions in Luria-Bertani (LB) broth, cation-adjusted Mueller-Hinton broth (CA-MHB) or trypticase soy broth (TSB) at 37 °C. LB broth (Cat. No. CM0996B), TSB, (Cat. No. CM0129B), and CA-MHB (Cat. No. CM0405B) were obtained from Oxoid (Basingstoke, UK). L-NPDNJ, amikacin sulfate (Sigma-Aldrich, Milan, Italy; Cat. No. A2324), and tobramycin sulfate (Sigma-Aldrich, Milan, Italy; Cat. No. T1783) were dissolved in ultrapure water.

### 2.3 Antibacterial activity

#### 2.3.1 Determination of minimum inhibitory concentration (MIC) and minimum bactericidal concentration (MBC)

A microdilution method was used to assess the MIC values of L-NPDNJ against planktonic bacteria, in accordance with the guidelines recommended by the European Committee for Antimicrobial Susceptibility Testing (Eucast) of the European Society of Clinical Microbiology and Infectious Diseases (Escmid) (2020). Briefly, serial dilutions of L-NPDNJ (0.5–1024 μg/mL) in CA-MHB were dispensed into 96-well plates, containing 5 × 10^5^ colony-forming unit (CFU) of bacterial cell, and incubated at 37 °C for 18–24 h. The negative controls contained only the compound in CA-MHB, while the positive controls contained only the bacteria without the compound. Then, the growth was measured spectrophotometrically, and the MIC was the lowest at which bacterial growth was inhibited. For MBC determination, aliquots from MIC wells were spot-plated on LB agar and incubated at 37 °C for 24 h; MBC was the lowest concentration capable of killing ≥99.9% of the initial inoculum (Esposito et al., 2019).

#### 2.3.2 Time-killing curve

The bacterial survival of *S. maltophilia* K279a in the presence of L-NPDNJ was monitored over time at concentrations corresponding to 0.5 × , 1 × , 2 × , and 4 × MIC (Esposito et al., 2020c). Exponentially growing cells were adjusted to approximately 4 × 10^6^ CFU/mL in CA-MHB supplemented with the indicated concentrations of L-NPDNJ and incubated at 37 °C with agitation (200 rpm). Untreated cultures were included as controls. Samples were withdrawn at selected intervals and serially diluted in sterile buffer prior to being spread onto TSA plates. Following incubation at 37 °C for 24 h, the CFU were quantified.

#### 2.3.3 Checkerboard assay

The combination effects of L-NPDNJ with amikacin or tobramycin against *S. maltophilia* strains were evaluated using a microbroth checkerboard assay (De Gregorio et al., 2020). Amikacin or tobramycin (0.5–1024 μg/mL) and L-NPDNJ (2–1024 μg/mL) in CA-MHB were dispensed along rows and columns of 96-well plates, respectively, followed by inoculation with 5 × 106 CFU/mL bacteria. Wells containing bacteria alone served as growth controls, while wells containing only broth served as sterility controls. The plates were then incubated at 37 °C for 18–24 h, and absorbance was measured at 595 nm. To assess the possible interaction between L-NPDNJ either amikacin or tobramycin, the FIC index was calculated as previously described (De Gregorio et al., 2020).

### 2.4 RNA purification and quantitative real-time PCR (qRT-PCR)

*S. maltophilia* K279a cells were grown in LB broth at 37 °C with shaking at 200 rpm until they reached an optical density (OD) at 600 nm of 0.4, then split into two tubes and incubated for 3 h with or without 128 μg/mL of L-NPDNJ (Esposito et al., 2020c). Total RNA from three independent cultures was extracted with TRIzol reagent (Sigma-Aldrich, Milan, Italy; Cat. No. T3934), quantified using a Nanodrop instrument (Nanodrop Technologies), reverse-transcribed using the QuantiTect Reverse Transcription Kit (QIAGEN, Hilden, Germany; Cat. No. 205311), and analyzed by qRT-PCR with SYBR Green (Applied Biosystems; Cat. No. 4309155) as previously described (Esposito et al., 2020c). Gene expression levels were normalized using the housekeeping gene *rpoB*. The primers for the qRT-PCR experiments are provided in the Supplementary Table S1.

### 2.5 Hydrophobicity assay

Bacterial cell surface hydrophobicity (CSH) was evaluated using the microbial adhesion to hydrocarbons (MATHs) method as previously described (Rosenberg et al., 1980) with minor modifications. Briefly, bacterial cultures, either untreated or treated with L-NPDNJ at concentrations ranging from 64 to 128 μg/mL, were adjusted to an OD600 of 1.0. Then, 3 mL of each bacterial suspension were mixed with 1 mL of *p*-xylene (Sigma-Aldrich, Milan, Italy; Cat. No. 134449), vortexed for 20 s, incubated for 1 h at room temperature and the aqueous phase absorbance at 600 nm was measured. The CSH percentage was calculated using the following formula: CSH (%) = [A0–A/A0] × 100, where A0 and A are the absorbance values of the aqueous phase before and after contact with *p*-xylene, respectively (Karbowiak et al., 2022).

### 2.6 Adhesion to abiotic surfaces

The impact of L-NPDNJ on bacterial adhesion to abiotic surfaces was evaluated using the crystal violet (CV) staining method (Ramos-Hegazy et al., 2020). A bacterial suspension (5 × 106 CFU/mL) in TSB with 0.5% glucose was added to flat-bottom 96-well plates, treated with L-NPDNJ (16–128 μg/mL) or left untreated, and incubated at 37 °C for 4 h. Then, the wells were rinsed with phosphate-buffered saline (PBS), stained with 0.1% CV, washed twice with PBS, and the adsorbed dye was solubilized with 96% ethanol. Then, absorbance was measured at 595 nm after 20 min. Adherence reduction was expressed as a percentage, calculated using the formula: Relative adherence = [(Ac–At)/Ac] × 100, where Ac represents the OD_595_ of the untreated control wells and At corresponds to the OD_595_ of the L-NPDNJ -treated samples. Wells containing untreated *S. maltophilia* K279a cells were used as controls and these were considered to have 100% adhesion to polystyrene microtiter plate.

### 2.7 Preformed biofilm eradication

Biofilms were formed by incubating *S. maltophilia* (5 × 106 CFU/mL) in TSB with 0.5% glucose in flat-bottom 96-well plates at 37 °C for 24 h, then treated with L-NPDNJ (4–128 μg/mL) for additional 24 h (Esposito et al., 2020c). Control wells, in which the bacteria were not exposed to the compound, were incubated with broth only. Biofilm biomass was assessed by CV staining as described above. Viability was measured by tetrazolium salt reduction (XTT) assay according to the manufacturer's instructions (Roche Diagnostics; Cat. No. 11465015001). After incubation at 37 °C in the dark, biofilm metabolic activity was quantified by measuring absorbance at 490 nm using a microplate reader.

### 2.8 Motility assays

Swimming, swarming, and twitching motility assays were carried out on 0.3%, 0.5%, and 1% CA-MH agar plates, respectively (Roscetto et al., 2012). *S. maltophilia* K279a was grown overnight in CA-MH medium at 37 °C for 24 h, with or without L-NPDNJ (64 or 128 μg/mL), and cultures were then adjusted to an OD600 of 1.0. Swimming and swarming plates were inoculated with 2 μL of bacterial cultures and incubated at 30 °C for 24 h. Twitching was assessed by inoculation of bacteria at the bottom of the plates and visualized using Coomassie Brilliant Blue staining at the agar-dish interface.

### 2.9 Secreted protease activity assay

The effect of L-NPDNJ on the activity of protease secreted by the *S. maltophilia* K279a strain was assessed using LB agar plates supplemented with 2% skim milk (Sigma-Aldrich, Milan, Italy; Cat. No. 70166; Molloy et al., 2019). *S. maltophilia* K279a was cultured in DMEM, either with or without the addition of L-NPDNJ (64–128 μg/mL) for 24 h at 37 °C with agitation (200 rpm), and cultures were then adjusted to an OD600 of 0.6. After centrifugation at 12,000 x g for 15 min at 4 °C and filtration using 0.22 μm filters, 100 μL of cell-free supernatants (CS) was inoculated into an 8 mm diameter hole in the center of an agar plate. Proteolytic activity of CS was assessed after 48 h by measuring the clear zone diameter surrounding the hole (Kim et al., 2018).

### 2.10 A549 cell culture maintenance

A549 human lung epithelial cells (ATCC CCL 185) were cultured in 100 mm Petri plates with DMEM (Cytion GmbH, Berlin, Germany; Cat. No. 820300a) supplemented with 10% fetal bovine serum (FBS; Gibco, Thermo Fisher Scientific; Cat. No. A5670701), penicillin-streptomycin (10,000 U/mL; Gibco, Thermo Fisher Scientific; Cat. No. 15140122), and L-glutamine (2 mM; Gibco, Thermo Fisher Scientific; Cat. No. 25030081) at 37 °C in 5% CO_2_/95% air. Cells were detached using trypsin/EDTA solution (Gibco, Thermo Fisher Scientific; Cat. No. 25200056), collected in complete medium, centrifuged at 1200 rpm for 5 min, pellets were resuspended in fresh medium, properly diluted, and plated again.

### 2.11 Adhesion assay on A549 epithelial cells

A549 cells were grown as described above. *S. maltophilia* K279a cells were cultured up to an OD_600_ of 0.5 and then treated with or without 64 or 128 μg/mL of L-NPDNJ for 3 h. Bacteria were then washed twice and resuspended in DMEM (without FBS and penicillin/streptomycin) to a density of approximately 1 × 10^7^ CFU/mL (referred to as the starting bacterial CFU). A549 cells were infected with *S. maltophilia* K279a at an MOI of 50:1 for 1 h, washed 3 times with PBS and treated with 0.25% trypsin/EDTA. The cell-associated bacteria were quantified by plating serial dilutions on LB agar, and relative adherence was calculated using the formula: relative adherence = [adhered bacteria CFU of sample/ starting bacterial CFU of sample]/[adhered bacteria CFU of control/ starting bacterial CFU of control]. Controls were wells containing A549 cells infected with untreated *S. maltophilia* K279a cells and these were considered to have 100% adhesion A549 cells.

### 2.12 Evaluation of A549 cell layer integrity under exposure *S. maltophilia* K279a supernatants

The CS of *S. maltophilia* K279a was prepared as described above and diluted 1:8. A confluent monolayer of A549 cells was obtained by plating 10^5^cells/cm^2^ in 24 well plates and incubating at 37 °C and 5% CO_2_ in DMEM 10% FBS for 16–18 h. Prior to be treated, A549 cell were placed in serum-free DMEM for further 6 h, then exposed to either untreated or treated CS and acquired in time-lapse for 2,5 h at 37 °C and 5% CO_2_ by using a Zeiss Cell Observer system as previously described (Toscano et al., 2022). For data acquisition, the cell layers were sampled by acquiring phase contrast images (objective 10x) every 10 min for 2.5 h by using the setup developed as previously described (Toscano et al., 2024). The quantification of eukaryotic cell layer integrity has been obtained by adapting the procedure previously described (Agliarulo et al., 2015). Briefly the a-cellular area was quantified by using the wand tool in ImageJ (National Institute of Health, USA), with manual refinement of the selection in the presence of macroscopic errors. Every 30 min the integrity of the layer was assessed as the ratio between the total and the a-cellular area. The quantitative analysis of the cell-free area was derived from three independent experiments.

### 2.13 Macrophage phagocytosis and intracellular survival assay

RAW 264.7 macrophages (CLS Cell Lines Service) were grown at 37 °C in DMEM 10% FBS, 1% L-glutamine, 1% penicillin/streptomycin at 5% CO_2_. The macrophages were placed in 12-well plates at a density of 1 × 105 cells/well and left to adhere for 24 h. The cells were then infected with *S. maltophilia* K279a (treated with or without 64 or 128 μg/mL of L-NPDNJ for 3 h) at a MOI of 100 and incubated for 30 min at 37 °C and 5% CO_2_ (Roscetto et al., 2012). To remove extracellular bacteria, the cells were incubated for 1 h in DMEM supplemented with gentamicin (1 mg/mL), followed by three washes with PBS. We had previously established that gentamicin inhibits *S. maltophilia* K279a growth by 99.9% at this concentration. Afterwards, phagocytosed bacteria were quantified by lysing the macrophages and plating for viable bacterial counts. To measure intracellular survival, the RAW 264.7 cells were washed and incubated in freshly prepared medium containing a lower concentration of gentamicin (20 μg/mL) for 2 h and 24 h. At each time point, the macrophages were washed, lysed, and viable bacteria were calculated. Controls were wells containing cells infected with *S. maltophilia* K279a cells that had not been treated with L-NPDNJ and these were considered to have 100% phagocytosis or survival at 3 h or 24 h.

### 2.14 RAW 264.7 viability assay

RAW 264.7 cells were grown as described above. The macrophages were placed in 24-well plates at a density of 2 × 105 cells/well, incubated for 24 h, and then either untreated or pre-treated with L-NPDNJ (64 μg/mL) for 4 h. Cells were then infected with *S. maltophilia* K279a strain (either untreated or treated with 128 μg/mL of L-NPDNJ) at an MOI of 100 for 24 h at 37 °C in 5% CO_2_ (Nas et al., 2019). Macrophage viability was assessed using the 3-(4,5-dimethylthiazol-2-yl)-2,5-diphenyl-2H-tetrazolium bromide (MTT) assay. Briefly, MTT reagent (0.5 mg/mL; Sigma-Aldrich, Milan, Italy; Cat. No. M2128) was added to each well and the plate was incubated for 4 h at 37 °C. The formazan crystals were then dissolved in dimethyl sulfoxide, and the absorbance was measured at 570 nm. Controls were wells containing RAW 264.7 cells that had not been either treated with L-NPDNJ or infected with *S. maltophilia* K279a strain, and these were considered to have 100% viability.

### 2.15 *Galleria mellonella* killing assay

The virulence of *S. maltophilia* strains, both untreated and treated with L-NPDNJ, was evaluated by infecting *G. mellonella* larvae as previously described (Liu et al., 2018; Mamat et al., 2023). *S. maltophilia* K279a strain was cultured up to an OD_600_ of 0.5 in LB medium at 37 °C, then treated with or without 64 or 128 μg/mL of L-NPDNJ for 3 h, washed in PBS, and adjusted to 2 × 108 CFU/mL. Ten *G. mellonella* larvae (300–400 mg with no signs of melanization) were infected with 10 μl of the bacterial suspension (2 x 10^6^ CFU) through the left proleg using a Hamilton microliter syringe, after which the larvae were incubated in the dark at 37 °C in Petri dishes. This concentration has been previously determined to be the optimal dose of bacteria needed to kill the larvae within 48–96 h. PBS-injected larvae served as negative controls. The survival of the infected larvae was recorded at 24-h intervals over a period of 4 days. Larvae were classified as dead if they were unresponsive to touch and had fully melanized (blackening of the larvae). Experiments were performed in triplicate with at least three different batches of larvae, using at least four dilutions in each experiment. No ethical approval is required for the experimental use of *G. mellonella*.

### 2.16 Statistical analyses

All experiments were carried out in triplicate and repeated at least three times using three independent bacterial cultures. The results are shown as means ± standard deviation (SD). Data normality was assessed using the Shapiro-Wilk test. All datasets were normally distributed (*p* > 0.05), and parametric tests were therefore applied. The relative values were analyzed statistically using one-way ANOVA, followed by Dunnett's multiple comparison test, in GraphPad Prism version 8.0 (GraphPad Software, San Diego, CA). The Log-rank (Mantel-Cox) test was used to perform the statistical analysis of Kaplan-Meier survival curves, with GraphPad Prism version 8.0. The lethal doses 50% (LD_50_) values were analyzed using GraphPad Prism version 8.0.

## 3 Results

### 3.1 Synthesis of L-NPDNJ

**Figure 2:**
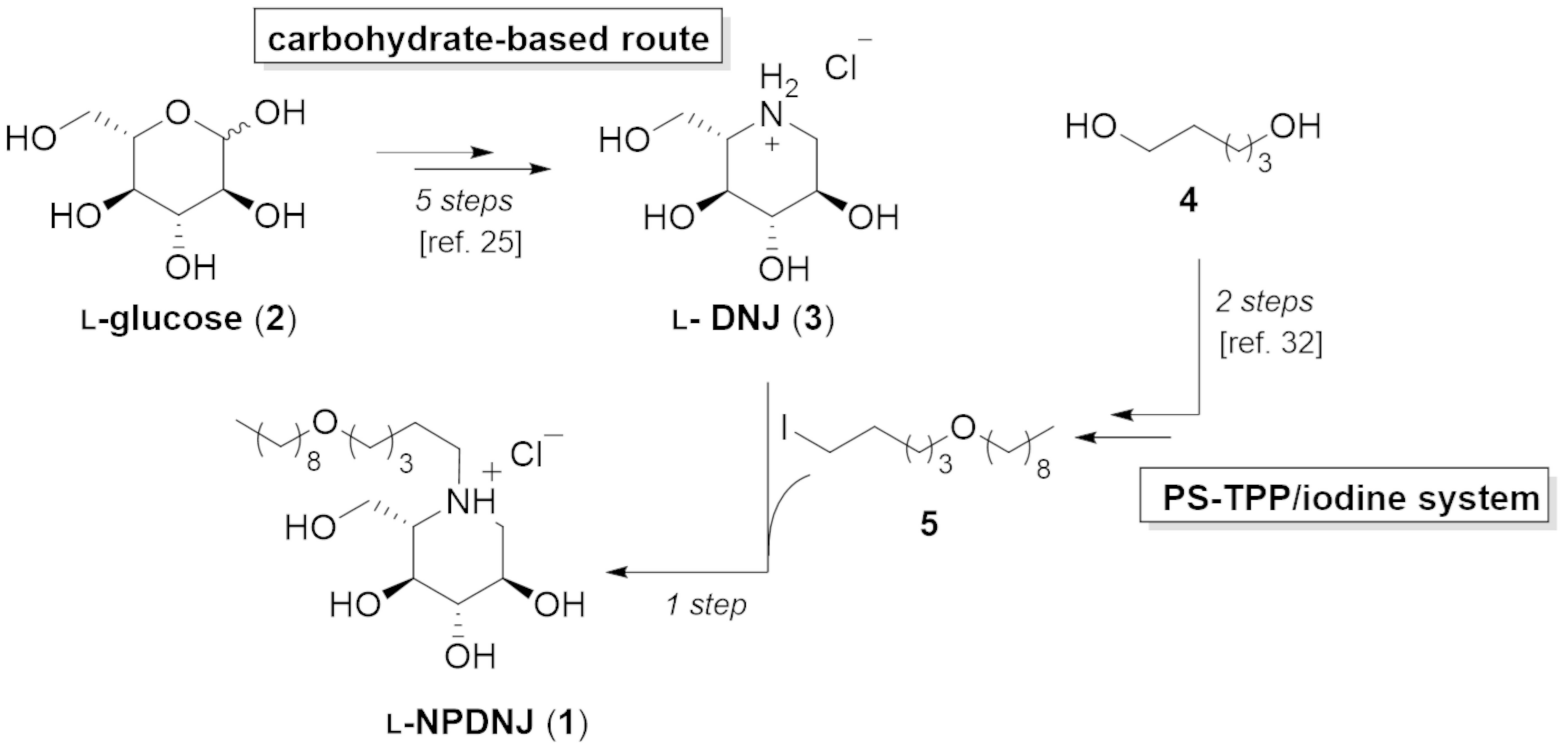
Scheme used for the synthesis of L-NPDNJ.

Although L-iminosugars have displayed very promising potential as therapeutic candidates they have been poorly explored due to the lack from natural sources and the limited availability of synthetic routes for their preparation. In this context we have recently prepared a series of *N*-substituted L-iminosugars, including L-NPDNJ. Our synthetic strategy relies on a carbohydrate-based route enabling the construction of the enantiopure L-DNJ (3) from the commercially available L-glucose (2), while *N*-functionalization of L-DNJ with alkoxyalkyl iodide 5 (in turn obtained by 1,5 pentanediol 4) to obtain the target L-NPDNJ (1) was achieved using a well-known synthetic protocol involving the polymer-supported triphenylphosphine (PS-TPP)/iodine activating system (Figure 2).

### 3.2 Evaluation of L-NPDNJ alone and combined with aminoglycosides against *S. maltophilia* strains

The MIC and MBC values of L-NPDNJ against 10 strains of *S. maltophilia* are reported in Supplementary Table S2. These include the reference strain K279a, which is used in laboratory research due to its complete genome, and nine clinical isolates. The results indicate that the antimicrobial activity of L-NPDNJ was relatively weak, as indicated by a MIC value of 128 μg/mL for the Sm0527, OBGtC9 and OBGtC13 isolates, and 256 μg/mL for the K279a strain and the remaining isolates.

**Figure 3:**
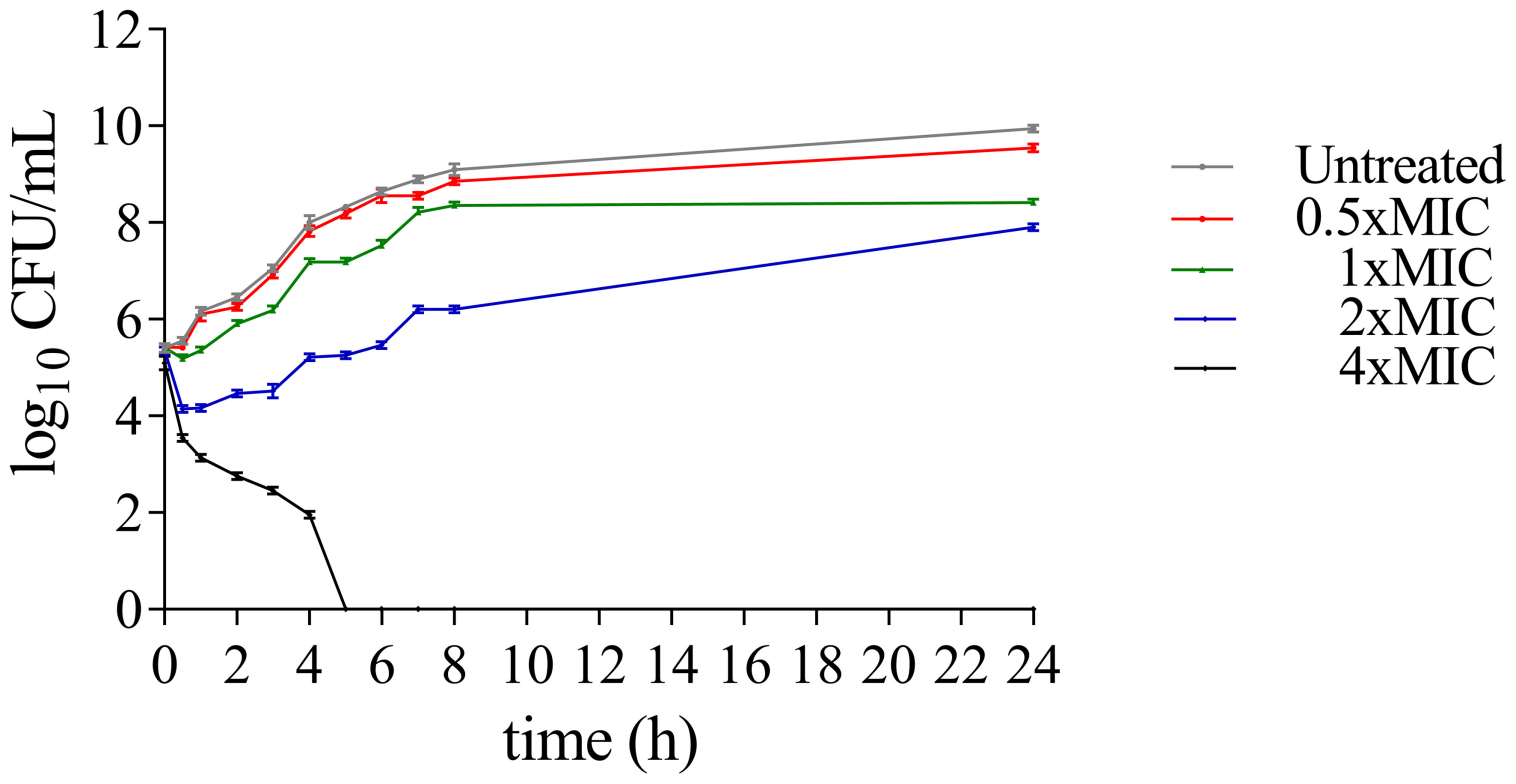
Killing curves for *S. maltophilia* K279a following treatment with the L-NPDNJ. Growth kinetics were measured after exposure to L-NPDNJ at 0.5 × MIC, MIC, 2 × MIC, and 4 × MIC. Data represent three independent biological replicates (*n* = 3), each performed in triplicate. The mean value is shown with ± SD.

The activity of L-NPDNJ against K279a strain was then assessed over time using a time-kill curve assay. This molecule exhibited concentration-dependent effects against *S. maltophilia* K279a (Figure 3). In the untreated control, bacterial numbers increased steadily over time, reaching approximately 6 x 10^9^ CFU/mL at 24 h, confirming robust bacterial growth in the absence of the compound. At a sub-inhibitory concentration of 0.5 × MIC and 1 × MIC values, L-NPDNJ exerted a modest inhibitory effect during the initial stages, delaying bacterial growth to a slight extent compared to the untreated control. However, bacterial counts increased over time, but a reduction in viable bacterial counts was observed after 24 h of exposure of bacteria to L-NPDNJ at both concentrations (3 x 10^9^ CFU/mL and 2.5 x 10^8^ CFU/mL, respectively). Treatment at 2 × MIC initially reduced the number of CFUs, followed by limited regrowth, resulting in lower bacterial loads compared with the lower concentrations. By contrast, exposure to 4 × MIC resulted in rapid and pronounced bactericidal activity. A marked decrease in viable counts was observed within the first 4–6 h, resulting in a reduction below the limit of detection, with no regrowth observed up to 24 h.

Combination therapy is a common treatment for clinically relevant infections and offers advantages over monotherapy, including broader antimicrobial coverage, reduced resistance development and toxicity, and, in some cases, synergistic activity that enhances pathogen clearance (Behera et al., 2025). Therefore, the synergistic potential of L-NPDNJ in combination with two aminoglycoside antibiotics, amikacin and tobramycin, was investigated using a checkerboard assay. MICs of the antibiotics and L-NPDNJ against *S. maltophilia* K279a and 545 strains were determined individually (MIC^a^) and in combination (MIC^c^), and the fractional inhibitory concentration index (FICI) was determined. As shown in Table 1, MIC value of L-NPDNJ alone was 256 μg/mL for both strains tested. The combination of either amikacin or tobramycin resulted in a 2- or 3-fold MIC reduction for *S. maltophilia* K279a and 545 strains, respectively, thus resulting in an additive effect for both strains (FICI = 0.625 and FICI = 0.750, respectively).

**Table 1:** Additive effects of the compound L-NPDNJ with aminoglicosides against *S. maltophilia* strains.

Strain	Combination	MIC^a^ (μg/mL)	MIC^c^ (μg/mL)	FICI
K279a	L-NPDNJ/amikacin	256/16	32/8	0.625
	L-NPDNJ/tobramycin	256/16	64/8	0.750
Sm0545	L-NPDNJ/amikacin	256/32	128/4	0.625
	L-NPDNJ/tobramycin	256/8	128/2	0.750

MIC^a^, MIC of one compound alone; MIC^c^, MIC of compounds used in combination; FICI, fractional inhibitory concentration index.

### 3.3 L-NPDNJ downregulates key virulence factors in *S. maltophilia* K279a

**Figure 4:**
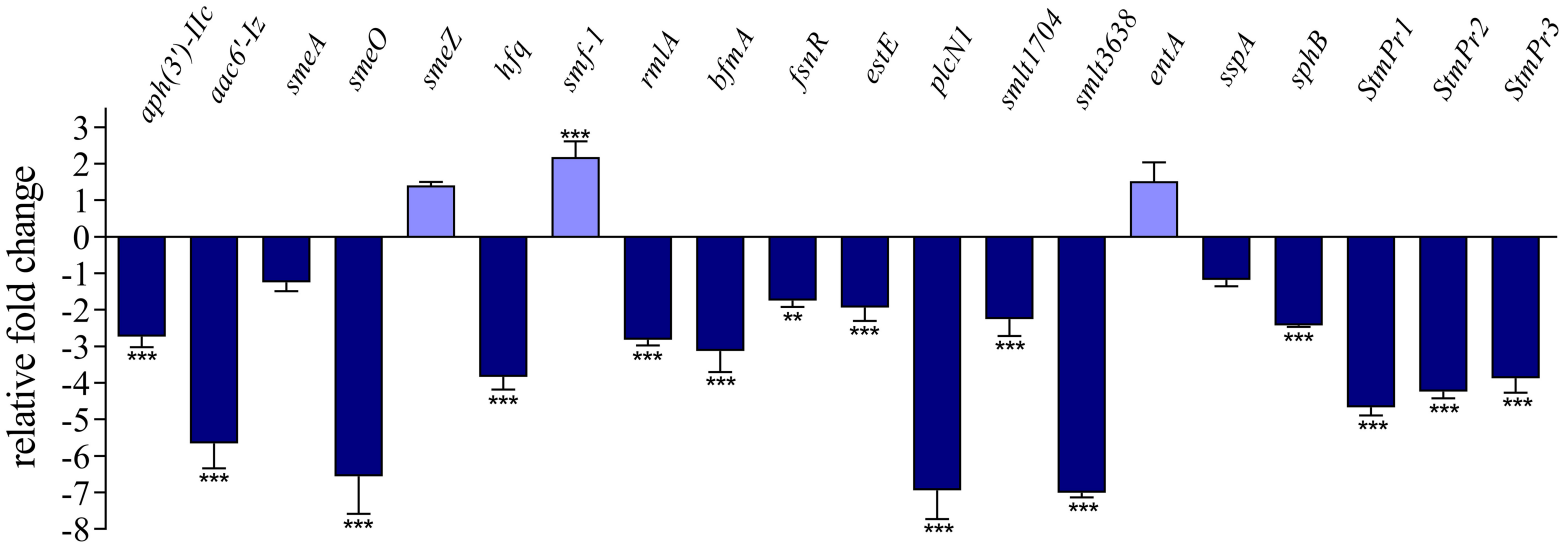
Transcriptional profiling of virulence factor genes in *S. maltophilia* K279a following treatment with L-NPDNJ determined by qRT-PCR. The *rpoB* expression was used to normalize the data. Data represent three independent biological replicates (*n* = 3), each performed in triplicate. Values are expressed as mean ± standard deviation (SD). Statistical analysis was performed using one-way ANOVA followed by Dunnett's multiple comparison test, with asterisks denoting significant differences between treated and untreated *S. maltophilia* cell (^**^ *p* < 0.005, ^***^ *p* < 0.001). *aac6-Iz*, aminoglycoside6′-N-acetyltransferase; *aph(3*′*)-IIc*, aminoglycoside3′-phosphotransferase; *bfmA*, two component response regulator; *entA*, putative enterobactin synthetase component A; *estE*, putative outer membrane esterase; *fsnR*, response regulator protein LuxR family; *hfq*, host factor-I protein; *plcN1*, putative non-hemolytic phospholipase C precursor; *rmlA*, glucose-1-phosphate thymidylyltransferase; *smeA*, drug resistance efflux protein; *smeO*, RND/Acr family transmembrane transporter; *smeZ*, multidrug ACR family efflux system; *smf-1*, fimbrial adhesin protein precursor; *smlt1704*, putative transmembrane CorC/HlyC family transporter; *smlt3638*, putative transmembrane hemolysin protein; *sphB*, putative autotransporter subtilisin-like protease; *spa*, putative protease IV; *stmPr1*, secreted serine protease; *stmPr2*, secreted serine protease; *stmPr3*, secreted serine protease.

In order to understand whether L-NPDNJ could serve as an anti-virulence agent, and to gain insight into its molecular effects on *S. maltophilia* K279a, quantitative real-time PCR was carried out. It was used to assess the expression of key genes involved in antibiotic resistance and bacterial virulence, such as those devoted to biofilm formation, and production of extracellular enzymes and global regulators. After 3 h of exposure to a sub-MIC concentration of L-NPDNJ, total RNA was extracted from K279a cells, while untreated cells cultured under the same conditions served as the control. No differences in growth were observed between treated and untreated cells in the experimental conditions utilized. The transcriptional profiles of L-NPDNJ-treated samples were compared with those of untreated controls, and the relative fold changes in gene expression were shown in Figure 4. In this experiment, only genes with relative transcript levels differing by at least 2-fold and a *p*-value < 0.001 were considered differentially expressed (see Supplementary Table S3). L-NPDNJ treatment resulted in a significant downregulation of genes associated with antibiotic resistance, including *aph(3*′*)-IIc, aac6-Iz*, and *smeO*, with reductions of approximately 2.73-, 5.66- and 6.56-fold, respectively. The expression of *hfq*, a pivotal post-transcriptional regulator involved in bacterial pathogenesis (Roscetto et al., 2012), was also moderately and significantly reduced following exposure to L-NPDNJ. Conversely, the expression of *smf-1*, which encodes a chaperone/usher pilus involved in biofilm formation and adhesion to cultured mammalian cells (Bhaumik et al., 2025), exhibited a significant slight increase in expression (+2.18-fold). Furthermore, L-NPDNJ treatment resulted in a significant downregulation of key genes involved in biofilm development, including *rmlA* and *bfmA* with reductions of approximately 2.82- and 3.13-fold, respectively. In addition to these regulatory effects, L-NPDNJ exposure also induced a significant inhibition of the expression of genes encoding extracellular enzymes. Specifically, the serine protease gene *sphB* was downregulated by 2.43-fold, while the major secreted protease genes *stmPr1, stmPr2*, and *stmPr3* were reduced by 4.67-, 4.24-, and 3.87-fold, respectively. Moreover, the decreased expression of *plcN1* (encoding a non-hemolytic phospholipase C), *smlt1704* (encoding a putative hemolysin), and s*mlt3638* (encoding a hemolysin III protein), suggests a possible reduced ability to lyse the host cells. Overall, these data indicate that L-NPDNJ affects the expression of genes involved in multiple virulence-associated pathways.

### 3.4 L-NPDNJ impairs cell surface hydrophobicity of *S. maltophilia*

**Figure 5:**
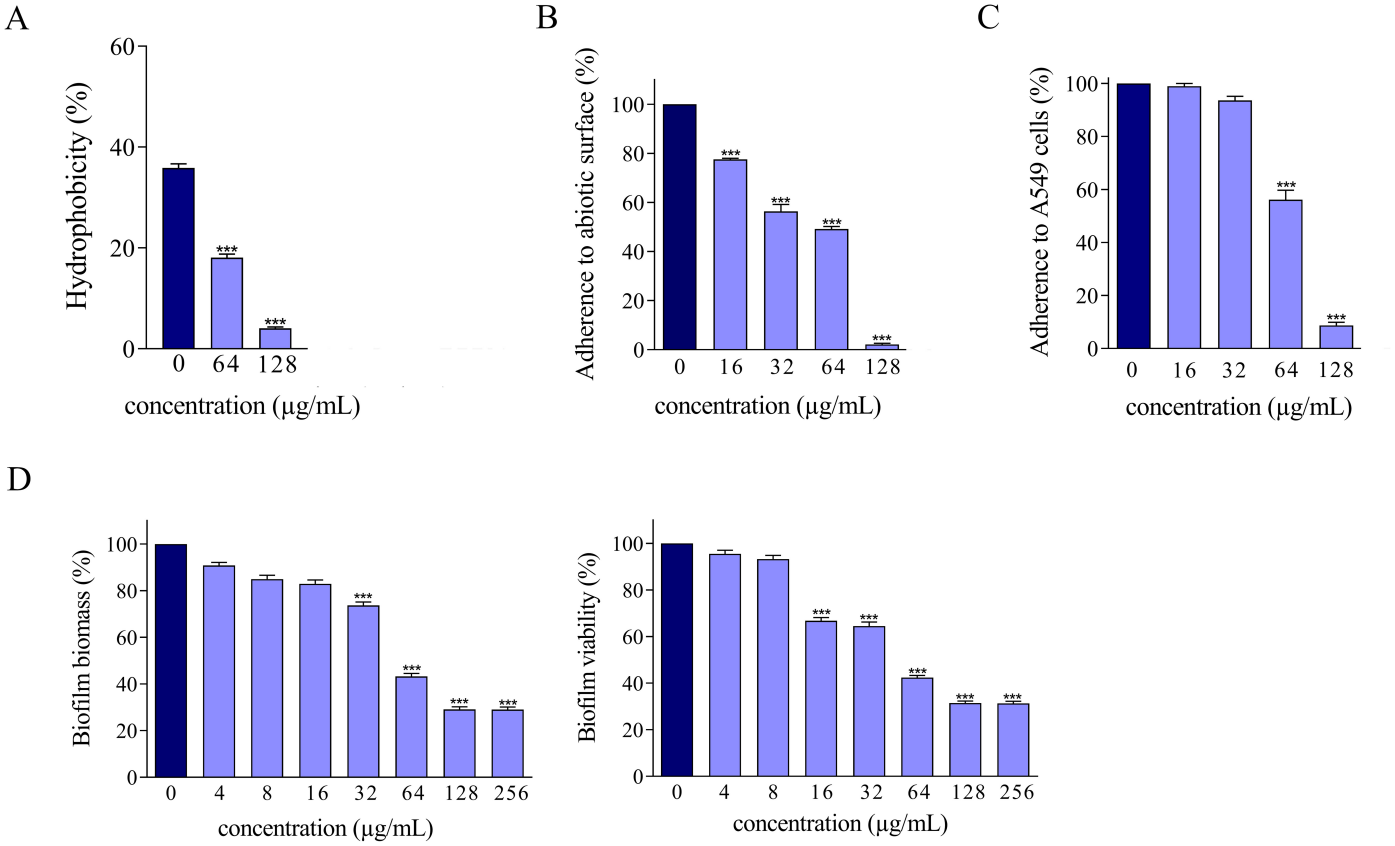
Effects of L-NPDNJ on cell surface hydrophobicity, abiotic and biotic adhesion, and preformed biofilm in *S. maltophilia* K279a. **(A)** Microbial adhesion to solvents. The percentage of hydrophobicity indicates percentage of adhesion to *p*-xylene solvent for *S. maltophilia* K279a untreated and treated with L-NPDNJ. **(B)** Adhesion *S. maltophilia* K279a untreated and treated with L-NPDNJ to polystyrene microplates. **(C)** Adhesion *S. maltophilia* K279a untreated and treated with L-NPDNJ to A549 human lung epithelial cells. **(D)** Eradicating effect of L-NPDNJ on *S. maltophilia* K279a preformed biofilm. *S. maltophilia* preformed biofilm was incubated with increasing L-NPDNJ concentrations for 24 h at 37 °C. The left panel showed biofilm biomass measured by CV staining. The right panel showed biofilm viability measured by XTT assay. Data represent three independent biological replicates (*n* = 3), each performed in triplicate. Values are expressed as mean ± SD. Statistical analysis was performed using one-way ANOVA followed by Dunnett's multiple comparison test, and statistically significant differences between the treated and untreated cells are indicated by asterisks (^***^p < 0.001).

Microbial CSH has been linked to characteristics associated with virulence, including adhesion, immune evasion and the development of biofilms (Krasowska and Sigler, 2014). In order to evaluate the effect of L-NPDNJ on CSH, *S. maltophilia* K279a cells were incubated with various concentrations of the compound and then hydrophobicity measured using the MATHs assay. Untreated K279a cells exhibited a CSH value of 36.6%, indicating a relatively hydrophobic surface (Figure 5A). Treatment with L-NPDNJ at concentrations of 64 μg/mL and 128 μg/mL led to a significant reduction in hydrophobicity, reaching 18.2% and 4.1%, respectively (Figure 5A). These results suggest that L-NPDNJ exposure leads to a dose-dependent reduction in surface hydrophobicity, potentially impairing bacterial adherence and virulence-related surface properties.

### 3.5 L-NPDNJ inhibits *S. maltophilia* adhesion to abiotic surfaces and to human lung epithelial cells

Bacterial adhesion to abiotic surfaces is a prerequisite for the development of biofilms and can facilitate the colonization of medical devices by *S. maltophilia*, thereby contributing to device-associated infections in humans (Lee et al., 2021). The effect of increasing concentrations (ranging from 16 μg/mL to 128 μg/mL) of L-NPDNJ on abiotic adhesion was evaluated by CV assay following 4 h of incubation at 37 °C. The results showed a concentration-dependent reduction of adhesion, with a 50% decrease observed at 32 μg/mL and a greater than 97% reduction at 128 μg/mL (Figure 5B). In order to exclude growth-dependent effects, planktonic growth was assessed under the same experimental conditions used for testing abiotic adhesion. No significant differences in bacterial growth were observed at any tested concentration, as shown by the time-killing experiment comparing untreated bacteria with those treated with 0.5 × MIC (128 μg/mL) L-NPDNJ at 4 h.

To investigate the effect of L-NPDNJ on bacterial adhesion to human lung epithelial cells, *S. maltophilia* K279a cells in the exponential phase were treated with L-NPDNJ at concentrations ranging from 16 μg/mL to 128 μg/mL for 3 h and then used to infect A549 human lung epithelial cells. The planktonic growth of K279a cells was not affected by L-NPDNJ at the concentrations that were tested in this assay. The number of cell-adherent bacteria was quantified and compared to that of the untreated control group. Treatment with L-NPDNJ resulted in a significant reduction in bacterial adhesion, with levels decreasing by 45% at 64 μg/mL and more than 90% at 128 μg/mL compared to untreated cells, respectively (Figure 5C).

### 3.6 L-NPDNJ eradicated preformed *S. maltophilia* biofilms

The ability of *S. maltophilia* to grow in biofilms on non-living surfaces and in host tissues has been linked to a high risk of pulmonary exacerbations and a decline in lung function (García et al., 2023). Furthermore, it acts as an alternative persistence strategy in strains that are more susceptible to antibiotic (Pompilio et al., 2020; Pompilio and Di Bonaventura, 2026). Indeed, the ability of L-NPDNJ to disrupt mature biofilms was evaluated by treating 24-h-old *S. maltophilia* biofilms for 18 h with L-NPDNJ (4–256 μg/ml). As shown in Figure 5D, both the biofilm biomass and the metabolic activity of the bacterial cells within the biofilm decreased in a dose-dependent manner. Particularly, a significant decrease in biofilm biomass was observed, with reductions of approximately 55% and 70% at concentrations of 32 μg/mL and 128 μg/mL, respectively (Figure 5D, left panel). A similar trend was observed in biofilm metabolic activity (Figure 5D, right panel), suggesting that L-NPDNJ is able to eradicate pre-formed biofilms at higher concentrations.

### 3.7 L-NPDNJ slightly affects swimming but not swarming or twitching motility in *S. maltophilia*

The effect of L-NPDNJ on the motility of *S. maltophilia* K279a was assessed using swarming, twitching, and swimming assays. No significant changes in swarming or twitching motility were observed under the tested conditions, whereas a modest reduction in swimming motility was observed in the presence of L-NPDNJ at a concentration of 128 μg/mL (Supplementary Figure S3).

### 3.8 L-NPDNJ inhibits secreted extracellular proteases and attenuates *S. maltophilia*-induced cytopathic effects in human lung epithelial cells

Transcriptional profiling of *S. maltophilia* K279a treated with L-NPDNJ revealed a significant downregulation of several genes associated with extracellular proteolytic activity, including *sphB, stmPr1, stmPr2*, and *stmPr3*. To determine whether this also resulted in a reduction of extracellular protease secretion in CS, functional assays using skim milk agar were performed. CS from untreated K279a cultures exhibited a robust proteolytic halo, indicating active secretion of extracellular proteases (Supplementary Figure S4). In line with the qRT-PCR results, treatment with L-NPDNJ at concentrations of 64 and 128 μg/mL led to a complete elimination of the proteolytic halo. As shown in (Supplementary Figure S4), an approximate 34.6 mm diameter proteolytic halo was observed for the untreated sample, while it was undetectable in the treated samples.

**Figure 6:**
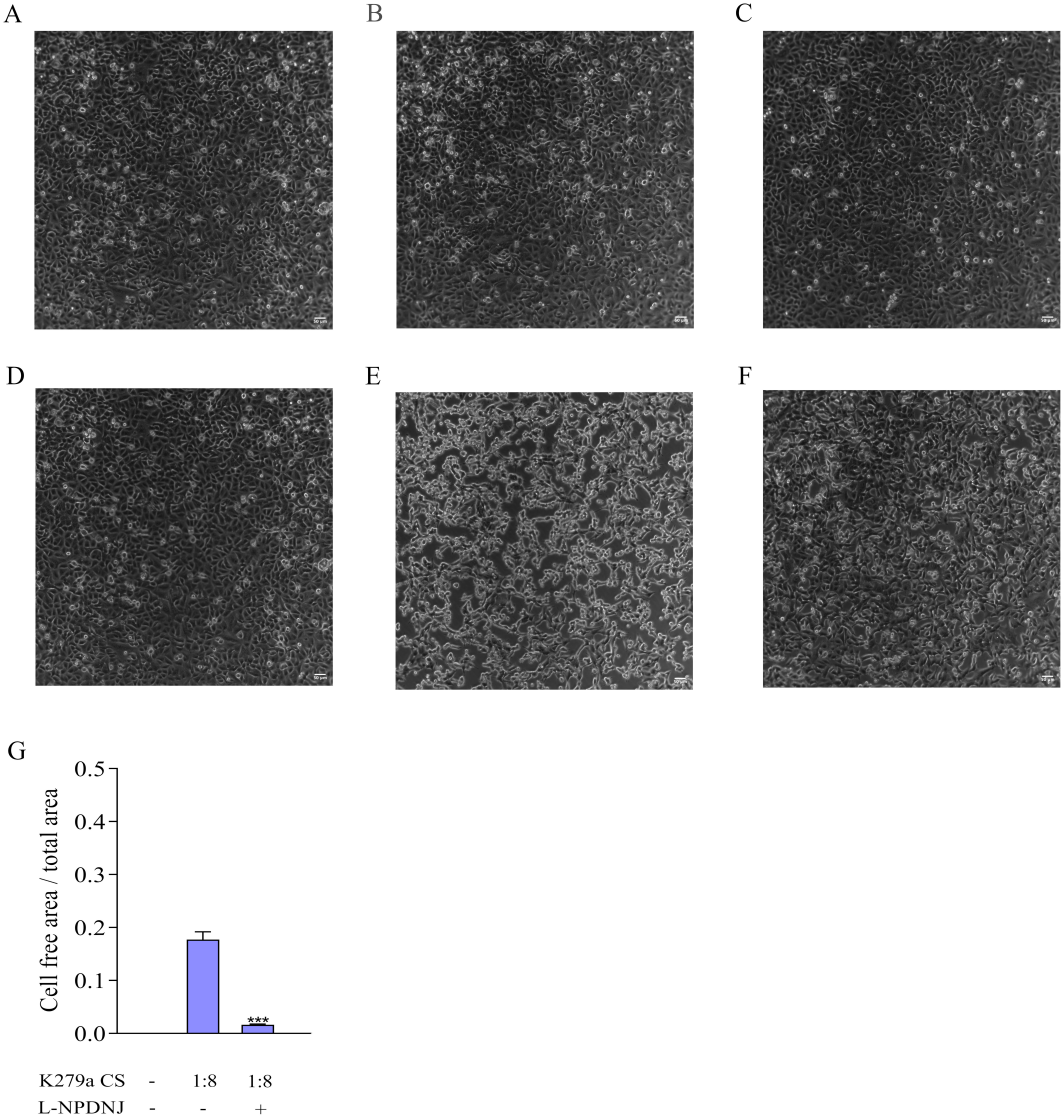
A549 cell layer integrity under exposure to K279a supernatants. Cells layers at T0 [top (**A**–**C**)] were obtained by plating 1 x 10^5^ cells/cm^2^ in DMEM supplemented with 10% FBS and incubated for16–18 h. Bottom panels depict same cell layers as above, after 150 min in free-serum DMEM with any treatment **(D)**, with a 1:8 dilution of CS from untreated K279a cells **(E)** or with a 1:8 dilution of CS from K279a cells treated with 128 μg/mL of L-NPDNJ **(F)**. The images are representative of three independent experiments. Representative phase contrast images (objective 10x) of different samples were acquired by using a Zeiss Cell Observer system. Acellular area quantification is reported in the graph in **(G)**. Data represent three independent biological replicates (*n* = 3). Values are expressed as mean ± SD. Statistical analysis was performed using one-way ANOVA followed by Dunnett's multiple comparison test, with asterisks denoting significant differences between acellular area of A549 cell layer treated with a 1:8 dilution of CS from K279a cells untreated and treated with 128 μg/mL of L-NPDNJ (^***^ *p* < 0.001).

As the extracellular proteases such as StmPr1 and StmPr2 are known to mediate *S. maltophilia*-induced cytopathic effects in A549 human lung epithelial cells (Karaba et al., 2013; DuMont et al., 2015; DuMont and Cianciotto, 2017), the ability of L-NPDNJ to attenuate this toxicity was subsequently assessed. A549 epithelial cell monolayers at 90% confluence (Figures 6A–C) were exposed for 150 minutes to CS obtained from *S. maltophilia* K279a untreated (Figure 6E) or treated with L-NPDNJ (Figure 6F). The morphological changes observed in the cells were then examined and compared with control cells that have not been exposed to bacterial CS (Figure 6D). In agreement with Molloy et al. (2020), exposure of A549 monolayers to CS from untreated K279a resulted in marked cytopathic changes, including the formation of cell-free areas (Figure 6E; see also Supplementary Video 1). By contrast, A549 cells exposed to CS from L-NPDNJ-treated bacteria exhibited preserved epithelial morphology and intact monolayers with minimal signs of cytotoxicity (Figure 6F; see also Supplementary Video 2), closely resembling the untreated control cells (Figure 6D; see also Supplementary Video 3). Quantification of cell-free areas showed that CS from untreated K279a cells induced a cell-free area of approximately 18% in confluent A549 monolayers. In contrast, CS from K279a cells treated with 128 μg/mL of L-NPDNJ reduced the cell-free area to less than 2% (Figure 6G).

### 3.9 L-NPDNJ stimulates macrophage phagocytosis and enhances macrophage intracellular clearance of *S. maltophilia* K279a

**Figure 7:**
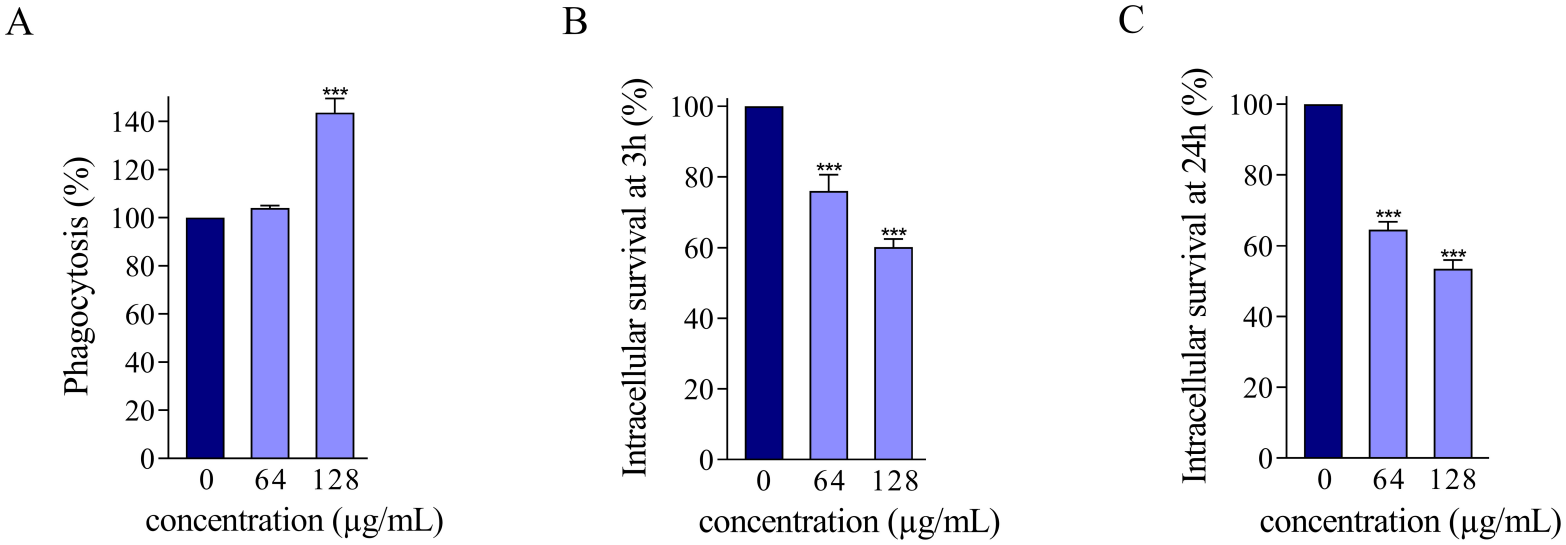
Effect of L-NPDNJ on *S. maltophilia* K279a phagocytosis and intracellular survival in macrophages. RAW 264.7 cells were infected with *S. maltophilia* K279a at an MOI of 100, either untreated or pre-treated with 64 or 128 μg/mL of L-NPDNJ for 3 h, and then incubated for 30 min. After 1 h treatment with gentamicin, the following were determined: phagocytosis **(A)**, intracellular survival in macrophages at 2 h **(B)**, and intracellular survival at 24 h post-infection **(C)**. Data represent three independent biological replicates (*n* = 3), each performed in triplicate. The mean value is shown with ± SD. Statistical analysis was performed using one-way ANOVA followed by Dunnett's multiple comparison test, with asterisks indicating statistically significant differences between treated and untreated bacterial cells (^***^p < 0.001).

To assess the impact of L-NPDNJ on host innate immune responses, the interaction between macrophages and *S. maltophilia* was evaluated. Exponentially growing *S. maltophilia* K279a cells were left untreated or exposed to L-NPDNJ (at concentrations of either 128 μg/mL or 64 μg/mL) for 3 h and then employed to infect RAW 264.7 macrophages. Phagocytosis assays revealed an increase in bacterial uptake in response to L-NPDNJ treatment. Although a slight effect was observed at 64 μg/mL, a significant 45% increase was obtained at 128 μg/mL compared to the untreated control group (Figure 7A). Furthermore, the results of intracellular survival assays showed that L-NPDNJ significantly diminished bacterial persistence within macrophages. At 3 h post-infection, the pretreatment of *S. maltophilia* K279a cells with L-NPDNJ at concentrations of 64 and 128 μg/mL reduced the percentage of living bacteria inside macrophages by 24% and 40%, respectively, compared to the total number of internalized bacteria (Figure 7B). This effect was sustained at 24 h, with intracellular survival decreased by 35% and 46%, respectively (Figure 7C).

### 3.10 L-NPDNJ enhances RAW 264.7 macrophage survival

**Figure 8:**
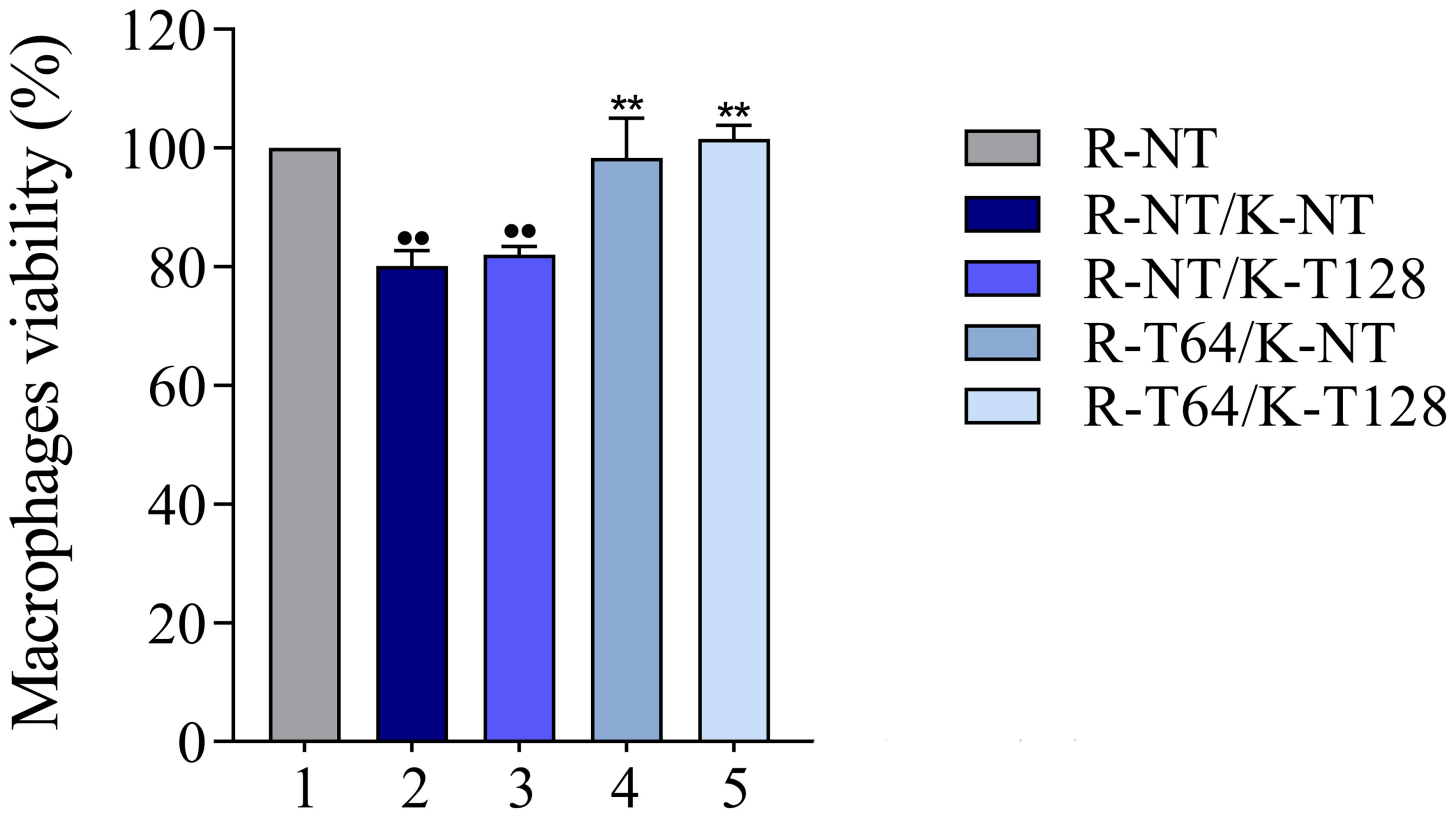
Effect of L-NPDNJ on viability of RAW 264.7 cells following infection with *S. maltophilia* K279a. Macrophage monolayers were either left uninfected (R-NT) or treated with 64 μg/mL of L-NPDNJ (R-T64), then infected with K279a cells that were either untreated (K-NT) or treated with 128 μg/mL of L-NPDNJ (K-T128). After 24 h of incubation, the number of viable macrophages was measured using an MTT assay. Data represent three independent biological replicates (*n* = 3), each performed in triplicate. The mean value is shown with ± SD. Statistical analysis was performed using one-way ANOVA followed by Dunnett's multiple comparison test, with asterisks denoting statistically significant differences between samples (••*p* < 0.01 vs R-NT and ^**^p < 0.01 vs. R-NT/ K-NT).

*S. maltophilia* can induce apoptosis in macrophages, thereby facilitating immune evasion and intracellular survival (Nas et al., 2019). To assess the ability of L-NPDNJ to modulate this host-pathogen interaction, RAW 264.7 macrophages were either left untreated or pre-treated with L-NPDNJ, then infected with *S. maltophilia* K279a cells that had either been left untreated or pre-treated with L-NPDNJ. 24 h after infection, the macrophage viability was assessed using the MTT assay. RAW cells that had not been either treated with L-NPDNJ or infected with the *S. maltophilia* K279a strain were considered to be 100% viable (Figure 8, sample 1). Untreated *S. maltophilia* K279a cells had a detrimental effect on the survival rates of untreated macrophages (80%, Figure 8, sample 2). Pre-treatment of the bacteria with L-NPDNJ did not enhance the survival of untreated macrophages (81%, sample 3 in Figure 8). On the contrary, pre-treatment of macrophages with L-NPDNJ significantly increased cell viability upon infection with untreated bacteria (96%, sample 4 in Figure 8). Furthermore, when both macrophages and bacteria were treated, survival increased to a rate of 100% (Figure 8, sample 5).

### 3.11 Virulence attenuation of *S. maltophilia* K279a by L-NPDNJ treatment in the *G. mellonella* infection model

**Figure 9:**
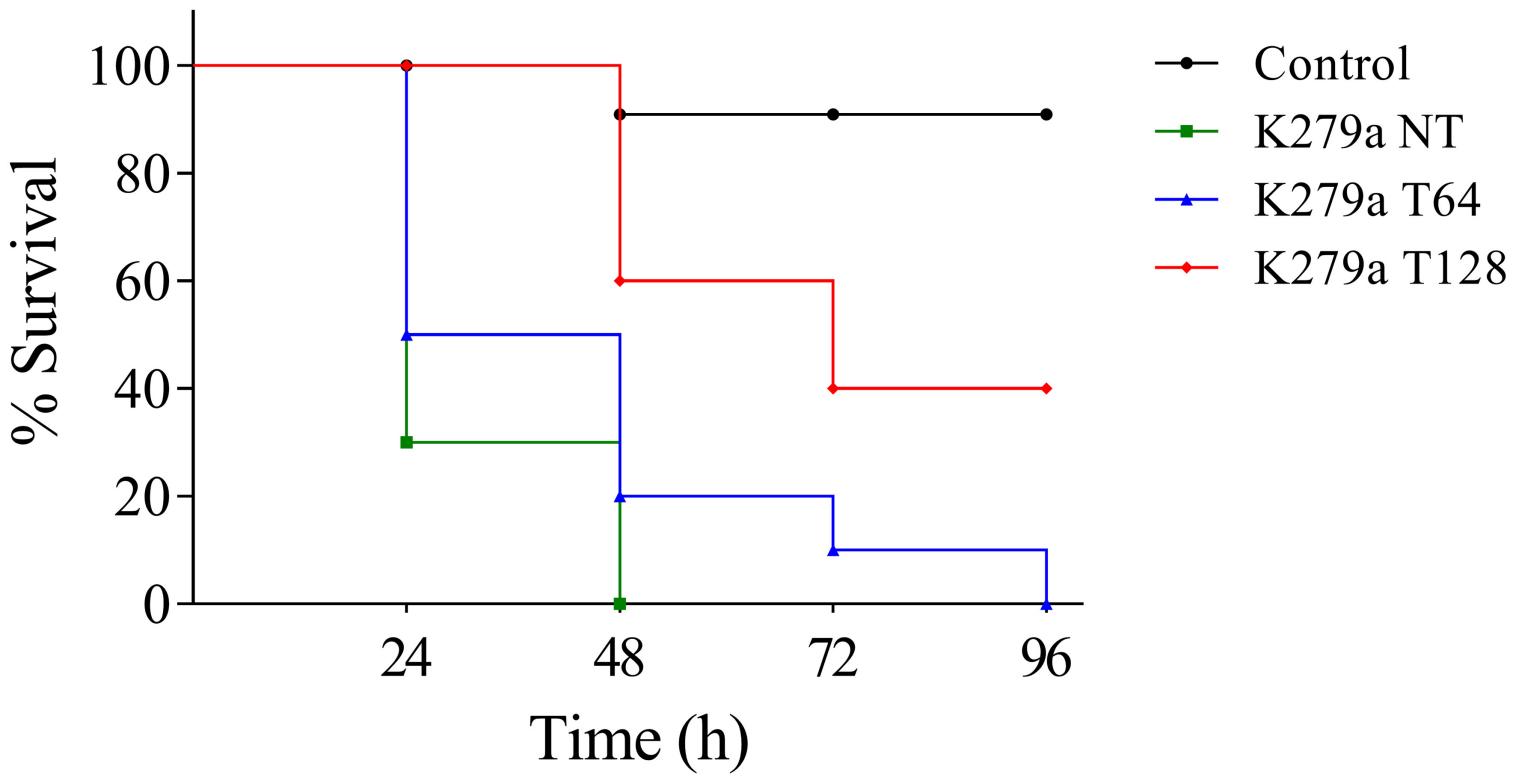
Kaplan–Meier survival curves of *G. mellonella* larvae infected with K279a untreated or pretreated with L-NPDNJ. Larvae (*n* = 10 per group) were infected with 10 μl of K279a cells either untreated (NT) or pretreated with L-NPDNJ 64 μg/mL (T64) or 128 μg/mL (T128), respectively. Larvae infected with 10 μl of PBS were considered as control. Experiments were performed in triplicate with at least three different batches of larvae, using at least four dilutions in each experiment. The Log-rank (Mantel-Cox) test was used to perform the statistical analysis of Kaplan-Meier survival curve.

*G. mellonella* is a reliable infection model for assessing the virulence of various bacterial species, including *S. maltophilia* (Alio et al., 2020). This infection model was used to evaluate the effectiveness of L-NPDNJ in reducing the production and release of virulence factors *in vivo*, by comparing the virulence of untreated and L-NPDNJ-pretreated *S. maltophilia* K279a strains. The infective dose used (approximately 2 x 106 CFU per larva) was previously established as the optimal bacterial concentration capable of inducing larval mortality within 48–96 h, thus providing a robust dynamic range for assessing differences in virulence. In line with this, larvae infected with untreated K279a cells exhibited rapid mortality, with 100% lethality observed within 48 h of infection (Figure 9). Conversely, larvae infected with bacteria pretreated with L-NPDNJ exhibited markedly higher survival rates. At 48 h post-infection, approximately 60% and 20% of larvae infected with K279a pretreated with 128 μg/mL and 64 μg/mL of L-NPDNJ, respectively, were still alive (Figure 9). At 96 h, the survival rate of larvae infected with K279a pretreated with 128 μg/mL of L-NPDNJ remained at 40%, while larvae infected with K279a pretreated with 64 μg/mL of L-NPDNJ exhibited complete mortality (Figure 9). Therefore, larvae infected with untreated K279a cells died much faster as compared to those infected with 128 μg/mL of L-NPDNJ K279a pretreated and survival curves of larvae infected with K279a pretreated with 128 μg/mL were significantly different (*p* < 0.001). Also, the median LD_50_ values for untreated K279a and for K279a pretreated with 64 μg/mL and 128 μg/mL of L-NPDNJ were about 1 × 10^4^, 1 × 10^5^ and 1 × 10^6^ cells, respectively. These results indicate a dose-dependent attenuation of the virulence of *S. maltophilia* K279a, as a consequence of treatment with L-NPDNJ.

## 4 Discussion

The increasing threat posed by multidrug-resistant (MDR) pathogens, such as *S. maltophilia*, necessitates the investigation of alternative therapeutic strategies beyond conventional antibiotics (Mojica et al., 2022). In this context, anti-virulence strategies emerged as a promising alternative by targeting pathogenic traits without exerting strong selective pressure for resistance (Dehbanipour and Ghalavand, 2022). As previously reported, the unnatural L-iminosugars could represent an attractive new tool in anti-virulence therapy (De Gregorio et al., 2020). The data collected herein demonstrate that the iminosugar L-NPDNJ, despite its weak antibacterial activity, exerts a broad-spectrum anti-virulence effect against *S. maltophilia*. These effects include reduced adhesion to host cells, impaired biofilm formation, decreased secretion of extracellular enzymes, and attenuation of cytotoxicity and immune evasion. Indeed, antimicrobial susceptibility testing confirmed the limited activity of L-NPDNJ against *S. maltophilia* (Supplementary Table S2). However, when evaluated in combination with aminoglycosides such as amikacin and tobramycin, L-NPDNJ exhibited additive effects, reducing MICs by 2- to 3-fold (Table 1). Aminoglycosides are an important class of drugs for tackling drug-resistant Gram-negative pathogens (Lang et al., 2023). The World Health Organization (WHO) now classifies them as a critically important class of antibiotics [World Health Organization (WHO), 2019], and they are still widely used in the empirical and definitive treatment of a broad range of infections. As demonstrated by Chuchalin et al. (2009), inhalation of tobramycin has been shown to enhance lung function substantially and reduce the burden of *P. aeruginosa* in CF patients, and it is now regarded as a standard therapeutic approach. Furthermore, *S. maltophilia* is frequently co-isolated with *P. aeruginosa* in CF patients, providing a strong clinical rationale for the selection of tobramycin in checkerboard assays. The enhanced activity of aminoglycosides in combined L-NPDNJ treatments may be attributed to the ability of the compound to modulate bacterial resistance pathways. Specifically, transcriptional profiling by qRT-PCR revealed significant downregulation of multiple genes associated with aminoglycoside resistance, including *aph(3)IIc, aac(6)-Iz*, and *smeO* (Figure 4). The resistance of *S. maltophilia* to aminoglycosides is contributed by the expression of different aminoglycoside-modifying enzymes, as well as the activation of multiple efflux pumps (Brooke, 2021). The aminoglicoside-modifying enzymes conferring resistance to this class of antibiotics include O-phosphotransferases (APHs) and N-acetyltransferases (AACs) (Mojica et al., 2022). For instance, AAC (6)-Iz confers reduced susceptibility to amikacin, netilmicin, sisomicin, and tobramycin (Li et al., 2003). Furthermore, L-NPDNJ was found to downregulate *smeO*, a component of the SmeOP-TolCSm efflux system. This RND-type multidrug efflux pump has been demonstrated to mediate the extrusion of a wide array of substrates, including antibiotics such as amikacin, gentamicin, and doxycycline (Lin et al., 2014). Suppression of this efflux system may enhance the intracellular accumulation of aminoglycosides, thereby contributing to the observed additive effects. Taken together, these findings suggest that L-NPDNJ could modulate bacterial resistance mechanism. Because these observations are based on transcriptional changes and additive effects in checkerboard assays, further studies are required to determine whether L-NPDNJ directly affects aminoglycoside susceptibility mechanisms.

A key finding of this study is that L-NPDNJ exposure is associated with transcriptional changes in several *S. maltophilia* genes linked to bacterial virulence. qRT-PCR analysis revealed a significant downregulation of genes implicated in virulence regulation, including *rmlA, bfmA*, and *hfq* (Figure 4). These genes are involved in processes fundamental to bacterial pathogenicity, such as LPS/EPS biosynthesis, envelope integrity, adhesion to abiotic and biotic surfaces and biofilm development. Indeed, RmlA (glucose-1-phosphate thymidylyltransferase), an essential enzyme in the biosynthesis of the O-antigen, has been demonstrated to be associated with biofilm formation, bacterial adhesion, and twitching motility (Huang et al., 2006). Furthermore, the transcription factor BfmA has been implicated in swimming motility and biofilm formation (Zheng et al., 2016). Conversely, the RNA-binding protein Hfq is crucial for *S. maltophilia* to adhere to epithelial cells and persist in macrophages (Roscetto et al., 2012). Our data are in agreement with previous studies indicating that the expression of *bfmA* gene is affected by *S. maltophilia* treatments with celastrol (Kim et al., 2018) or PYED-1 (Esposito et al., 2020c).

In addition to its impact on virulence-associated regulatory genes, L-NPDNJ was able to suppress the expression of several effector genes (Figure 4), encoding secreted proteases (e.g., *stmPr1-3, sphB*, and *sspA*) and phospholipases (*plcN1*). These enzymes play a critical role in host tissue degradation, inflammation, immune evasion, and cytotoxicity (Mikhailovich et al., 2024). *S. maltophilia* clinical isolates produce high levels of phospholipases, which contribute to pathogenesis by destroying cell membranes and/or inducing an intense inflammatory response (Bhaumik et al., 2024). Proteolytic activity of *S. maltophilia* has been associated with fulminant hemorrhagic pneumonia (Gutierrez et al., 2016) and with lung tissue damage in CF patients infected with this organism (Molloy et al., 2020). In particular, the serine proteases StmPr1, StmPr2 and StmPr3 have been shown to facilitate host tissue injury and immune modulation. These effects are mediated through cytoskeletal alterations, degrading extracellular matrix proteins in lung epithelial cells, promoting cell detachment, and degrading interleukin-8 (Windhorst et al., 2002; DuMont et al., 2015; DuMont and Cianciotto, 2017). As demonstrated in Supplementary Figure S4, functional assays confirmed that L-NPDNJ completely inhibited proteolytic activity, indicating that its effects extend beyond transcriptional regulation to the functional impairment of secreted virulence factors. In line with this observation, exposure of A549 monolayers to culture supernatants derived from L-NPDNJ-treated *S. maltophilia* K279a led to an attenuation of cytopathic effects (Figure 6F). Given that epithelial injury is largely mediated by the secreted serine proteases StmPr1, StmPr2, and StmPr3 (Windhorst et al., 2002; DuMont and Cianciotto, 2017; Molloy et al., 2020), the reduced formation of cell-free areas in the A549 monolayer observed in this study is likely attributable to decreased release of extracellular proteases. Additional studies are required to establish a direct correlation between transcriptional changes, protease production, and subsequent host-cell damage. In light of the well-established role of bacterial proteases in mediating host tissue damage and immune modulation, the inhibition of protease activity further reinforces the potential use of L-NPDNJ as a strategy to mitigate *S. maltophilia* pathogenicity without directly targeting bacterial viability.

It is well established that sub-inhibitory concentrations of antimicrobial agents can modulate a wide range of bacterial cellular functions, including adhesion, CSH, motility, biofilm and interactions with the host, such as phagocytosis and phagocyte-mediated killing (Dehbanipour and Ghalavand, 2022). In line with these observations, L-NPDNJ induced marked alterations in bacterial cell surface properties, as evidenced by a significant reduction in microbial CSH (Figure 5A). CSH is a key factor in bacterial attachment to epithelial cells and plays an important role in immune evasion uptake by immune cells (Krasowska and Sigler, 2014). In *S. maltophilia*, the observed decrease in CSH was found to be associated with impaired adhesion to both abiotic and biotic surfaces. This finding is consistent with previous results by Pompilio et al. (2008), showing that *S. maltophilia* strains with higher CSH levels exhibited enhanced adhesion to abiotic surfaces, increased biofilm formation, and reduced swimming motility, while twitching motility remained unaffected.

Notably, the slight upregulation of *smf1* observed under the tested conditions does not result in an increased adhesion phenotype. We speculate that increased expression of *smf1* is a compensatory transcriptional response that is insufficient to counterbalance the impairment of other adhesion-related pathways; additionally, post-transcriptional or post-translational regulatory mechanisms may further limit the assembly and production or functionality of fimbrial structures.

Furthermore, L-NPDNJ demonstrated a notable antibiofilm activity against mature biofilms, which reduced biofilm biomass and metabolic activity by 70% at the highest tested concentration (Figure 5D). However, further analyses are needed to distinguish between biofilm matrix disruption and reduction in viable biofilm-associated bacteria. Previous studies have shown that alterations in bacterial cell surface properties can have a significant impact on the cell-cell adhesion and structural integrity of *S. maltophilia* biofilms (Di Bonaventura et al., 2004). The biofilm eradicating activity exerted by L-NPDNJ is of particular clinical relevance, as biofilm structures hinder the efficacy of antibiotics by limiting drug diffusion and enhancing antimicrobial inactivation. Concurrently, biofilms protect embedded bacteria from host immune defenses acting as a physical barrier and impairing local phagocytosis (Campoccia et al., 2019).

Treatment with L-NPDNJ was also found to influence host-pathogen interactions by reducing epithelial cell adhesion (Figure 5C), and by enhancing bacterial uptake and intracellular clearance of *S. maltophilia* by RAW 264.7 murine macrophages (Figure 7). This could be attributed, at least in part, to increased bacterial hydrophilicity, which facilitates phagocytosis (da Silva Domingues et al., 2013), as well as to the downregulation of virulence factors that normally protect the bacteria from host-mediated killing. Although the contribution of CSH to phagocytosis appears to vary among different bacterial species (Jankute et al., 2017; Kado et al., 2019; May et al., 2019), our data support a model in which reduced CSH in *S. maltophilia* is associated with attenuated virulence and enhanced immune clearance.

In addition, our data demonstrated that L-NPDNJ directly improved macrophage viability upon bacterial infection (Figure 8). As demonstrated in earlier studies, *S. maltophilia* has been found to employ a type IV secretion system to elicit pro-apoptotic effects in macrophages, thus enabling immune evasion (Nas et al., 2019). While the treatment with L-NPDNJ of bacteria alone did not rescue host cell survival, pre-treatment of macrophages with L-NPDNJ conferred a significant protection against *S. maltophilia*-induced cytotoxicity. These results suggest that L-NPDNJ may exert a direct effect on host cells modulating cellular responses involved in survival processes When both macrophages and bacteria were treated, an additive protective effect was observed (Figure 8), indicating that L-NPDNJ not only reduces bacterial virulence but also reinforces host cell resilience. In addition to L-NPDNJ bacterial anti-virulence activity, the potential contribution of host-directed mechanisms should be also taken into account in the overall interpretation of these findings. Further studies specifically addressing the direct impact of L-NPDNJ on host cell viability and activation in the absence of infection are necessary to clarify these aspects.

Crucially, the anti-virulence effect of L-NPDNJ was confirmed *in vivo* using the *G. mellonella* infection model (Figure 9). This model has previously been validated for the study of the pathogenesis and treatment of *S. maltophilia* infections (Li et al., 2023). Larvae infected with *S. maltophilia* K279a pretreated with L-NPDNJ exhibited a significant, dose-dependent enhancement in survival, with protective effects sustained up to 96 h post-infection. These *in vivo* findings are consistent with the transcriptional and phenotypic data reported above, including the downregulation of protease-encoding genes, reduced extracellular enzyme secretion, and preserved epithelial cell integrity. Further studies are needed to determine the therapeutic potential of this compound *in vivo*. These studies will require the direct administration of L-NPDNJ to infected larvae and animal models, including prophylactic and post-infection treatment regimens, as well as host toxicity assessments.

In conclusion, this study reveals that L-NPDNJ is a potential anti-virulence agent capable of targeting multiple virulence determinants of *S. maltophilia* without affecting bacterial viability. It also demonstrates the possible effectiveness of combining L-NPDNJ with other drugs against multidrug-resistant *S. maltophilia* strains. Furthermore, L-NPDNJ has been shown to exert both pathogen-directed and host-modulating effects, although the relative contribution of each component remains to be fully elucidated.

The following limitations are identified with this study. The analyses were performed using only the *S. maltophilia* K279a reference strain. Given the genetic and phenotypic heterogeneity of *S. maltophilia* strains, it remains to be established whether similar effects occur in clinically relevant isolates. Also, because the specific bacterial targets and molecular mechanisms are still unclear, the observed effects may be dependent both on the interference in carbohydrate-related and cell envelope-associated processes. Further research is required to characterize the bacterial pathways and molecular networks affected by L-NPDNJ, using transcriptomic and proteomic analyses, as well as efflux-related assays or mutant-based approaches.

## Data Availability

The datasets presented in this study can be found in online repositories. The names of the repository/repositories and accession number(s) can be found in the article/Supplementary material.
