# Supplementary material for: N-nonyloxypentyl-L-deoxynojirimycin reduces Stenotrophomonas maltophilia virulence in vitro and in Galleria mellonella infection model
Source: Front Microbiol. 2026 May 29;17:1835243. doi: 10.3389/fmicb.2026.1835243 (PMC13260231; doi:10.3389/fmicb.2026.1835243)
Supplement: Supplementary file 1 [file Data_Sheet_1.docx]

Supplementary Material

# 1. Supplementary Data

**Chemistry**

1. ***Chemical Synthesis***

l-NPDNJ has been synthesized by our established procedure starting from l-glucose. NMR spectra, optical rotation value and purity (determined by qHNMR) were fully in agreement with those previously reported by us (<https://doi.org/10.1021/acs.jmedchem.2c01617>).

^1^H NMR (400 MHz, CD_3_OD) δ: 4.10 (d, 1H, *J* = 12.3 Hz), 3.88 (dd, 1H, *J* = 2.9, 12.3 Hz), 3.78 −3.68 (ddd, 1H, *J* = 4.6, 9.3, 11.5 Hz), 3.58 (dd, 1H, *J* = 9.6, 10.4 Hz), 3.48-3.40 (m, 5H), 3.39−3.32 (m, 2H), 3.24−3.11 (m, 1H), 3.03 (bd, 1H, *J* = 9.6 Hz), 2.98 (t, 1H, *J* = 11.5 Hz), 1.88−1.70 (m, 1H), 1.69−1.60 (m, 2H), 1.58−1.50 (m, 2H), 1.49−1.41 (m, 2H), 1.38−1.21 (m, 12H), 0.88 (t, 3H, *J* = 6.2 Hz). ^13^C NMR (100 MHz, CD_3_OD) δ: 78.2, 72.2, 71.4, 68.7, 67.8, 67.3, 55.0, 54.9, 54.3, 33.0, 30.8, 30.7, 30.6, 30.4, 30.1, 27.3, 24.5, 23.9, 23.7, 14.4. [α]_D_ +10.0, c 0.53, MeOH.

# 2. Supplementary Figures and Tables

## 2.1 Supplementary Figures

**Figure S1.** ^1^H NMR spectrum of l-NPDNJ (400 MHz, CD_3_OD).

**Figure S2.** ^13^C NMR spectrum of l-NPDNJ (100 MHz, CD_3_OD).

**Figure S3.** Effect of l-NPDNJ on *S. maltophilia* motility. Representative images of the swimming motility formed by strain K279a after 24 h incubation period at 30 °C on 0.3% CA-MH agar plates. (A) untreated control (NT); (B) treated with 64 µg/mL l-NPDNJ; (C) treated with 128 µg/mL l-NPDNJ.


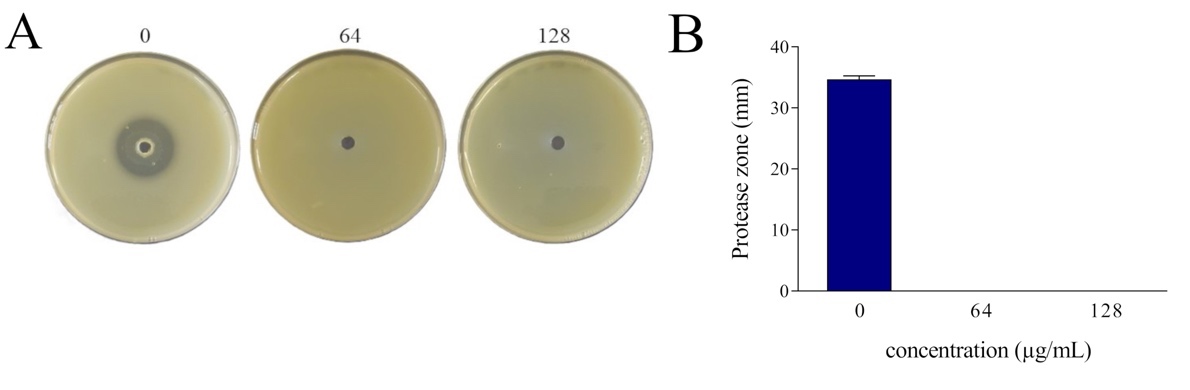


**Figure S4.** Effect of l-NPDNJ on extracellular proteases in *S. maltophilia* K279a. Secreted protease assays were performed using culture supernatants. Proteolytic activity was determined using LB agar containing 2% skim milk (A) and diameter of transparent zones surrounding the holes was measured at 48 h (B). Experiments were carried out in triplicate and repeated 3 times. The mean value is shown with SD.

**2.2 Supplementary Videos**

**Supplementary Movie 1**: The movie reports the phase contrast images (objective 10x) of A549 cell layers incubated with a 1:8 dilution of CS from untreated K279a cells, acquired in time-lapse every 10 minutes for 2.5 h.

**Supplementary Movie 2**: The movie reports the phase contrast images (objective 10x) of A549 cell layers incubated with a 1:8 dilution of CS from K279a cells treated with 128 mg/mL of l-NPDNJ, acquired in time-lapse every 10 minutes for 2.5 h.

**Supplementary Movie 3**: The movie reports the phase contrast images (objective 10x) of A549 cell layers incubated in free-serum DMEM with any treatment, acquired in time-lapse every 10 minutes for 2.5 h.

## 2.3 Supplementary Tables

**Table S1.** MIC and MBC values of *N*-substituted l-NPDNJ against *S. maltophilia* K279a reference strain and *S. maltophilia* clinical isolates.

| **Strain** | **l-NPDNJ** | |
| --- | --- | --- |
|  | **MIC (μg/mL)** | **MBC (μg/mL)** |
| K279a | 256 | 1000 |
| Sm0262 | 256 | 512 |
| Sm0527 | 128 | 256 |
| Sm0545 | 256 | 512 |
| Sm0571 | 256 | 512 |
| Sm0781 | 256 | 1000 |
| OBGtC9 | 128 | 256 |
| OBGtC13 | 128 | 256 |
| OBGtC22 | 256 | 256 |
| OBGtC28 | 256 | 512 |

**Table S2.** RT-PCR analysis of biofilm and virulence factors gene expression in *S. maltophilia* K279a in the presence of l-NPDNJ.

| **Genes** | **Description** | **Fold change^a^** ± **SD** | **p-values** |
| --- | --- | --- | --- |
| *aph(3')-IIc* | aminoglycoside 3' phosphotransferase | -2.73 ± 0.291 | <0.001 |
| *aac6'-Iz* | aminoglycoside 6'-N-acetyltransferase | -5.66 ± 0.676 | <0.001 |
| *smeA* | drug resistance efflux protein | -1.25 ± 0.230 | 0.73 |
| *smeO* | RND/Acr family transmembrane transporter | -6.56 ± 1.025 | <0.001 |
| *smeZ* | multidrug ACR family efflux system | +1.41 ± 0.098 | 0.14 |
| *hfq* | host factor-I protein | -3.84 ± 0.347 | <0.001 |
| *smf-1* | fimbrial adhesin protein precursor | +2.18 ± 0.432 | <0.001 |
| *rlmA* | glucose-1-phosphate thymidylyltransferase | -2.82 ± 0.155 | <0.001 |
| *bfmA* | two component response regulator protein | -3.13 ± 0.573 | <0.001 |
| *fsnR* | response regulator protein LuxR family | -1.75 ± 0.164 | 0.003 |
| *estE* | outer membrane esterase | -1.94 ± 0.365 | <0.001 |
| *plcN1* | non-hemolytic phospholipase C precursor | -6.95 ± 0.782 | <0.001 |
| *smlt 1704* | transmembrane CorC/HlyC family transporter | -2.26 ± 0.455 | <0.001 |
| *smlt 3638* | transmembrane hemolysin protein | -7.01 ± 0.128 | <0.001 |
| *entA* | enterobactin synthetase component A | +1.52 ± 0.519 | >0.99 |
| *sspA* | protease IV | -1.18 ± 0.173 | 0.26 |
| *sphB* | autotransporter subtilisin-like protease | -2.43 ± 0.032 | <0.001 |
| *StmPr1* | secreted serine protease | -4.67 ± 0.220 | <0.001 |
| *StmPr2* | secreted serine protease | -4.24 ± 0.177 | <0.001 |
| *StmPr3* | secreted serine protease | -3.87 ± 0.397 | <0.001 |

**^a^** -indicates reduction and + indicates increase.

**Table S3.** Genes analyzed and oligonucleotide sequences used in this study.

| **Gene ID** | **Gene name** | **Description** | **Primer name** | **Primer sequence** | **Reference** |
| --- | --- | --- | --- | --- | --- |
| Smlt3615 | *aac6′-Iz* | putative aminoglycoside 6'-N-acetyltransferase | aac6-Iz fw | TGTGGACTGATGCCGATG | (56) |
|  |  |  | aac6-Iz rv | GCACTTCAGCGAAACCAAC |  |
| Smlt2120 | *aph(3’)-IIc* | putative aminoglycoside 3'-phosphotransferase | aph3-IIc fw | CCGATCATGAAGACCTGGTG | (56) |
|  |  |  | aph3-IIc rv | GTCGATGAAACCGCTGAAAC |  |
| Smlt4209 | *bfmA* | putative two component response regulator | bfmA fw | AGTGAACTGCGCTTTTCTGG | (56) |
|  |  |  | bfmA rv | TGAATTCACCACGGCTGAG |  |
| Smlt2817 | *entA* | putative enterobactin synthetase component A | entA fw | TGATCGTTACCGTCAGTTCG | This study |
|  |  |  | entA rv | ACGATGTTGCAGCGGATG |  |
| Smlt3773 | *estE* | putative outer membrane esterase | estE fw | TCGATGGTTACACCGAAAGC | This study |
|  |  |  | estE rv | ACGGTCGAATCAACGTTCTG |  |
| Smlt2299 | *fsnR* | putative response regulator protein LuxR family | fsnR fw | TCCTGATGGACCTGTCATTG | (56) |
|  |  |  | fsnR rv | TGCATGGTCATCATCACAAC |  |
| Smlt1736 | *hfq* | host factor-I protein | hfq fw | TCTACAAGCACGCCATTTCC | (56) |
|  |  |  | hfq rv | TACTCGTCTGCTTCATCACCTG |  |
| Smlt1755 | *plcN1* | putative non-hemolytic phospholipase C precursor | plcN1 fw | TCAGTACCGAGGGCGAATAC | This study |
|  |  |  | plcN1 rv | CACGGCGAGATCACATACAG |  |
| Smlt0648 | *rmlA* | putative glucose-1-phosphate thymidylyltransferase | rmlA fw | TGCTGGGTGACAACATCTTC | (56) |
|  |  |  | rmlA rv | CCGGATCATTCACCCAATAG |  |
| Smlt0898 | *rpoB* | putative DNA-directed RNA polymerase, beta-subunit | rpoB fw | AGGAAATGCTGACGGTGAAG | (56) |
|  |  |  | rpoB rv | ACGAGCACGTTGAAGGATTC |  |
| Smlt4476 | *smeA* | putative drug resistance efflux protein | smeA fw | ATACGGCACTGACCACCATC | This study |
|  |  |  | smeA rv | TTTACCTGTGCCTTGCCATC |  |
| Smlt3925 | *smeO* | putative RND/Acr family transmembrane transporter | smeO fw | AACTGGACGTGGCTGACTTC | This study |
|  |  |  | smeO rvv | TGGTAGCGCTTCAGGTCATC |  |
| Smlt2202 | *smeZ* | putative multidrug ACR family efflux system | smeZ fw | GCAGTGATGTACCTGTTTCTGC | (56) |
|  |  |  | smeZ rv | CAGCACATTGATCGAGAAGC |  |
| Smlt0706 | *smf-1* | putative fimbrial adhesin protein precursor | smf-1 fw | ACCGTGTCCAAGAACACTCTG | (56) |
|  |  |  | smf-1 rv | TGCACTTGGTCAGGTTGATG |  |
| Smlt1704 |  | putative transmembrane CorC/HlyC family transporter | Smlt1704 fw | AAATCATGGCCGAACACG | This study |
|  |  |  | Smlt1704 rv | AGCGATTTCAGTTCGAGCAC |  |
| Smlt3638 | - | putative transmembrane hemolysin protein | Smlt3638 fw | GGTTGAAGGTATTCGACCACTG | (56) |
|  |  |  | Smlt3638 rv | ATCAGGGTGAACGGGGTATAG |  |
| Smlt3524 | *sphB* | putative autotransporter subtilisin-like protease | sphB fw | CGCATCTTTCAGTCACCAAC | (55) |
|  |  |  | sphB rv | GTAATTGAAGTTGGCCAGCAC |  |
| Smlt4190 | *sppA* | putative protease IV | sppA fw | AGTTTCTTCATCGGGCTGTG | (55) |
|  |  |  | sppA rv | ATGACGAACATCACCAGCAG |  |
| Smlt0686 | *stmPr1* | putative secreted serine protease | StmPr1 fw | GCCGAAGTCATCAACCTCTC | (55) |
|  |  |  | StmPr1 rv | ACACGTTGGTGTTGCTGTTG |  |
| Smlt0861 | *stmPr2* | putative secreted serine protease | StmPr2 fw | GCCGAGATCATCAACATGAG | This study |
|  |  |  | StmPr2 rv | GTTGGCCACATTCATCGAG |  |
| Smlt4395 | *stmPr3* | putative secreted serine protease | StmPr3 fw | ATCGACAGCACCTGCAACTAC | (55) |
|  |  |  | StmPr3 rv | TTCACATCGCGATAGGACAG |  |
